# Effectiveness, safety, and immunogenicity of half dose ChAdOx1 nCoV-19 COVID-19 Vaccine: Viana project

**DOI:** 10.3389/fimmu.2022.966416

**Published:** 2022-08-29

**Authors:** Valéria Valim, Olindo Assis Martins-Filho, Maria da Penha Gomes Gouvea, Luiz Antônio Bastos Camacho, Daniel Antunes Maciel Villela, Sheila Maria Barbosa de Lima, Adriana Souza Azevedo, Lauro Ferreira Pinto Neto, Carla Magda Allan Santos Domingues, Nésio Fernandes de Medeiros Junior, Isac Ribeiro Moulaz, Laiza Hombre Dias, Samira Tatiyama Miyamoto, Andréa Teixeira-Carvalho, José Geraldo Mill, Half Dose ChAdOx Study Group, Thayná Martins Gouveia

**Affiliations:** ^1^ Hospital Universitário Cassiano Antônio Moraes [HUCAM-UFES/ Empresa de Serviços Hospitalares (EBSERH)] and Programa de Pós Graduação em Saúde Coletiva (PPGSC), Universidade Federal do Espírito Santo, Vitória, Brazil; ^2^ Grupo Integrado de Pesquisas em Biomarcadores, Instituto René Rachou, Fundação Oswaldo Cruz (FIOCRUZ-Minas), Belo Horizonte, Brazil; ^3^ Escola Nacional de Saúde Pública, Fundação Oswaldo Cruz (FIOCRUZ), Rio de Janeiro, Brazil; ^4^ Programa de Computação Científica (PROCC), Fundação Oswaldo Cruz (FIOCRUZ), Rio de Janeiro, Brazil; ^5^ Laboratório de Tecnologia Virológica (LATEV) (Bio-Manguinhos), Fundação Oswaldo Cruz (FIOCRUZ), Rio de Janeiro, Brazil; ^6^ Escola Superior de Ciências da Santa Casa de Misericórdia de Vitória (EMESCAM), Vitória, Brazil; ^7^ External Consultant, Independent Researcher, Vitória, Brazil; ^8^ Departamento de Educação Integrada em Saúde, Secretaria de Estado da Saúde do Espírito Santo (SESA), Vitória, Brazil; ^9^ Hospital Universitário Cassiano Antônio Moraes, Universidade Federal do Espírito Santo [HUCAM-UFES/ Empresa de Serviços Hospitalares (EBSERH)], Vitória, Brazil; ^10^ Departamento de Educação Integrada em Saúde, Universidade Federal do Espírito Santo (DEIS/UFES), Vitória, Brazil; ^11^ Hospital Universitário Cassiano Antônio Moraes [HUCAM-UFES/ Empresa de Serviços Hospitalares (EBSERH)] and Departamento de Ciências Fisiológicas, Universidade Federal do Espírito Santo, Vitória, Brazil

**Keywords:** COVID-19, fractional dose, ChAdOx1, effectiveness, immunogenicity, vaccine, safety

## Abstract

Fractional dose is an important strategy to increase access to vaccines. This study evaluated the effectiveness, safety, and immunogenicity of half dose of ChAdOx1 nCoV-19 vaccine. A non-inferiority non-randomized controlled trial compared a half dose of ChAdOx1 nCoV-19 with the full dose, with an interval of 8 to 10 weeks, in individuals aged 18–49 years. The primary endpoints were the incidence rate of new cases/1,000 person-year at 90 days after 14 days of the second dose, confirmed by RT-PCR and new cases registered at SUS National Health Surveillance Database (e-SUS VS). The anti-SARS-CoV-2 spike (S) protein receptor binding domain (RBD) by chemiluminescence and the neutralizing antibodies by plaque reduction neutralization test (PRNT) were titrated. The soluble biomarkers were quantified with a multiplex immunoassay. Follow-up was 90 days after 14 days of the second dose. A total of 29,598 individuals were vaccinated. After exclusion, 16,570 individuals who received half a dose and 6,402 who received full doses were analyzed. The incidence of new cases confirmed by RT-PCR of half dose was non-inferior to full dose (23.7 vs. 25.7 cases per 1,000 persons-year [coefficient group -0.09 CI95%(-0.49 to 0.31)], even after adjusting for age and sex. There were no deaths or hospitalization after immunization of either group. Immunogenicity was evaluated in a subsample (N=558) compared to 154 healthcare workers who received a full dose. The seroconversion rate in seronegative individuals at baseline half dose was 99.8%, similar to that of the full dose (100%). Geometric mean concentration (95% CI; BAU/mL) were half dose = 188 (163-217) and full dose = 529 (423–663) (p < 0.001). In seropositive subjects at baseline (pre-immune individuals), the first dose induced very high and similar IgG-S in half dose 1,359 (1,245-1,483) and full dose 1,354 (1,048–1,749) BAU/mL. A half dose induced a high increase in plasma chemokines, pro-inflammatory/regulatory cytokines, and growth factors. The frequency of adverse events was similar. No serious adverse events or deaths were reported. A half dose of ChAdOx1 nCoV-19 is as effective, safe, and immunogenic as the full dose. The immune response in pre-immune (seropositive in the baseline) individuals indicates that the half dose may be a booster dose schedule.

## 1 Introduction

The SARS-CoV-2 outbreak has prompted the rapid expansion of several technology platforms, such inactivated, viral vector-based, and mRNA vaccines (1, 2). Even when most of the American and European populations were already vaccinated, a new variant appeared on the African continent, which still has limited access to the vaccine, and it quickly spread to all continents. Another concern is that the immunity conferred by vaccines, as well the natural immunity conferred by new mutations, may require periodic booster doses in the coming years (2, 3).

Therefore, the scarcity of inputs for the production of vaccines on a large scale and at the necessary speed is a limitation for advancing worldwide vaccine coverage and guaranteeing booster doses (3). Vaccination with fractional doses is a strategy that has already been used to eliminate an outbreak of yellow fever in 2017 in Brazil and Africa (4). Alternative dosing has also been considered for seasonal influenza (5).

Existing evidence from clinical trials suggests that alternative doses of some vaccines could yield high immune responses, comparable to those for standard doses of the same vaccines and higher than those for some approved vaccines (6). A trial with mRNA-1273 (Moderna) found similar immune responses for both 50 and 100 µg (standard) doses (7). ChAdOx1 nCoV-19 was administered as a single- or two-dose regimen (28 days apart) with one of two formulations: 2.2×10^10^ virus particles (vp, low dose) or 3.5–6.5 × 10^10^ vp (which became the standard dose) in 560 volunteers. Within each age group, no significant differences were seen in neutralization titers between low- and standard-dose vaccine recipients (8).

An interim analysis of four randomized controlled trials in Brazil, South Africa, and the United Kingdom compared 4,440 individuals who received two standard doses (SD/SD) with 1,374 participants who received a low dose followed by a standard dose (LD/SD). Vaccine efficacy of the low dose (LD/SD) was 90.0%, which was higher than the efficacy of the standard dose (SD/SD) group (62.1%) (9). These results suggested that a half dose is immunogenic enough to confer protection against SARS-CoV-2. The aim of this study was to evaluate the effectiveness, safety, and immunogenicity of a half dose of ChAdOx1 nCoV-19.

## 2 Materials and methods

### 2.1 Study design

This was a non-inferiority non-randomized controlled trial that tested the fractional dose (half dose = 0.25 mL) of ChAdOx1 nCoV-19 (ChAd Half Dose) compared to the full dose (ChAd Full Dose) in adults from Viana city, Espírito Santo State, Brazil.

This study was developed by Universitary Hospital of Federal University of Espírito Santo (HUCAM-UFES/EBSERH), and it is registered at ClinicalTrials.gov (NCT05059106), and was approved by the National Research Ethics Committee (CONEP, Protocol No. 4.752.775/2021), and the Ethics Review Committee of the Pan American Health Organization (PAHOERC, Protocol No. 0367.02/2021).

### 2.2 Participants

All residents of Viana city, aged 18 to 49 years (estimated of 35,000), were eligible for inclusion in the study. The exclusion criteria were: pregnant women; history of severe allergic reaction (anaphylaxis) to any previously administered vaccine; having received another vaccine in the previous 14 days; fever or flu-like symptoms; previous vaccination against COVID-19 at any time; recent diagnosis of COVID-19 with onset of symptoms within 28 days before vaccination.

### 2.3 Recruitment

Participants were invited from meetings local leaders and authorities, and by a broad media campaign. The scheduling system was based on an online platform (www.vianavacinada.saude.es.gov.br). Viana residents interested in participating in the study and receiving the half dose, registered on the digital platform, up to the day before the vaccination, scheduled to take place on two pre-defined consecutive days. Among the first 15,000 registered, 600 (stratified by sex and age) were randomly invited to collect biological samples and directed by the system to the collection site. Of these 600 pre-registered, 558 attended and agreed to participate in the immunogenicity subgroup. Samples were collected on the same day and immediately before the first dose. Vaccination took place at the same collection site.

All participants provided online signed informed consent. This research was carried out using vaccines, health service, and information recording from the Brazilian National Public Health System (Unified Health System/SUS).

### 2.4 Study groups

**Figure 1:**
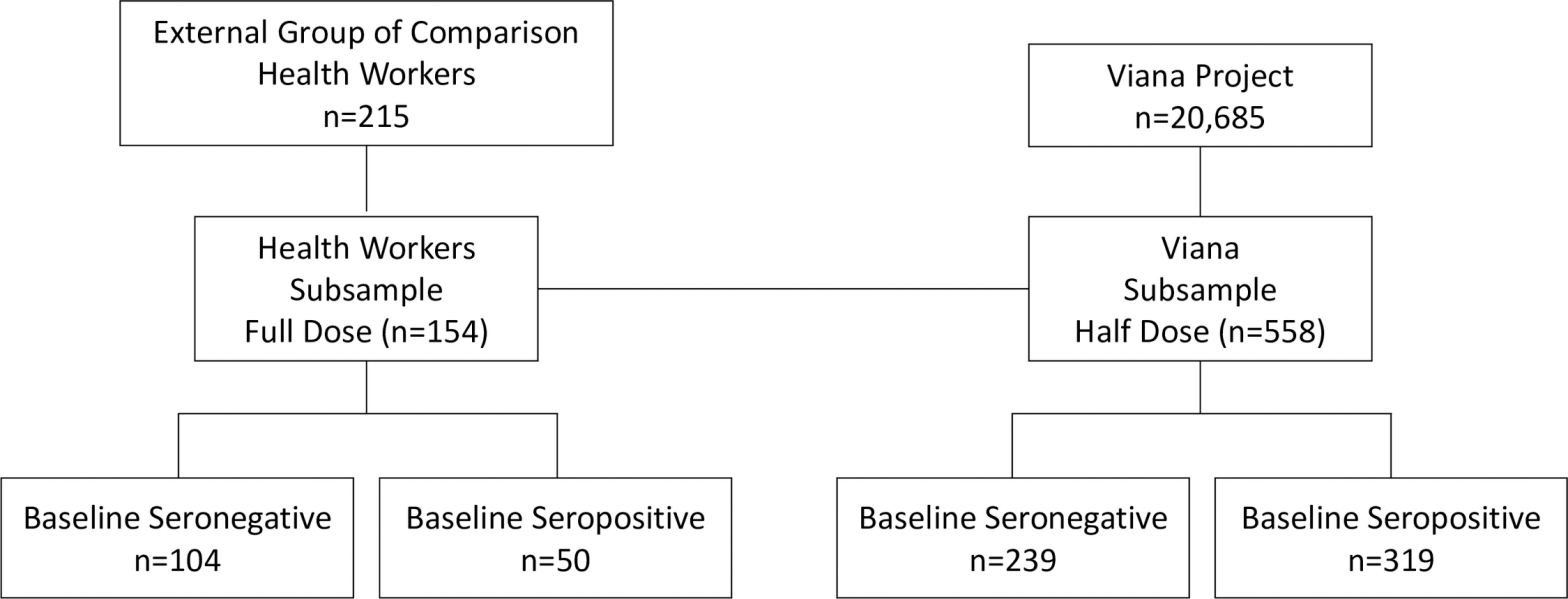
Flowchart of the immunogenicity study subsample.

Participants who received a half dose (ChAd Half Dose) were compared with those who received the full standard dose (ChAd Full Dose) (**Figure 1**). An age-stratified subsample of participants who received a half dose was randomly selected with a digital platform for blood collection to study humoral and cellular immune responses.

### 2.5 External group of comparison for immunogenicity outcomes

A subsample of healthcare workers from HUCAM-UFES/EBSERH, adjusted for age range, who received the full dose vaccine schedule of nCoV-19, was used to compare immunogenicity outcomes (Approval Ethics Committee HUCAM-UFES, No. 4.513.439/2021).

### 2.6 Procedures

#### 2.6.1 Intervention

Participants received ChAdOx1 nCoV-19 (AZD1222) vaccine, batches 215VCD130W (first dose), and 216VCD217W (second dose), production under technology transfer from AstraZeneca-Oxford to the Institute of Immunobiological Technology/BIO-MANGUINHOS/FIOCRUZ (Rio de Janeiro, Brazil). The immunization schedule was two doses by intramuscular injection, spaced 8 to 10 weeks apart, administered at primary care of the Brazilian National Public Health System (Unified Health System/SUS). Each 0.5 mL dose (full dose) contains 5×10^10^ viral particles from the replication-deficient recombinant chimpanzee adenovirus vector (ChAdOx1), which expresses the gene for the SARS-CoV-2 S protein. To minimize variation and increase precision, 1 mL syringes were used to inject 0.25 mL, which corresponded to half the standard dose.

The ChAd Half Dose and ChAd Full Dose groups were not randomized. The inclusion in each group was based on the participant’s decision, by voluntary adherence. Immunization schedules with half or full doses were available during the study inclusion period of 4 weeks in June 2021.

#### 2.6.2 Follow-up

Effectiveness outcomes (number of new cases, hospitalizations, and deaths) were evaluated 90 days after full protection, from day 14 after the second dose. Blood samples were collected to assess the humoral immune response before and 28 days after the first (D0 and D28) and second doses (D0* and D28*).

#### 2.6.3 Adverse events

Adverse events were secondary outcomes monitored by different strategies notifications in the SUS National System for Adverse Events (*e-SUS Notifica*); records of cell phone assistance were available for participants; electronic questionnaires were sent 7 and 28 days after the first and second doses by digital platform (http://www.vianavacinada.saude.gov.b); searching the service for customers´ support (SAC Fiocruz on 0800 021 0310) and Dial intoxications (0800 722 6001); evaluation of rumors that reached the state center for outbreak investigations (CIEVS 27-3636-8202 or duty 99849-1613), diary of adverse events in immunogenicity subsample participants; surveillance of all deaths of residents in Viana city.

Adverse events were assessed by a physician to establish severity and causality correlation (10), according to “Roadmap for Notification, Investigation, and Conclusion of Post-Vaccination Adverse Events” on *e-SUS Notifica*.

Guidelines on post-vaccination Adverse Event Surveillance, instructions for management, and adverse event care flow were published in technical notes (09/2021 – SESA/SSVS/GEVS/PEI, 10/2021 – SESA/SSVS/GEVS/PEI).

#### 2.6.4 Laboratory procedures

To assess the humoral immunogenicity in a subsample of participants, blood samples were collected through venipuncture at the cubital region, performed by an experienced professional. Ten milliliters of blood were collected in a tube with a separator gel to obtain serum for the evaluation of humoral reactivity.

The serum obtained from centrifuging the samples (1,300 g, for 15 min) was aliquoted and stored at -80°C in the Biorepository of the Clinical Research Center of HUCAM-UFES/EBSERH until further analysis.

Nasopharyngeal samples were collected in suspected cases to confirm SARS-CoV-2 by RT-PCR, and they were processed by the State Central Laboratory (LACEN, Vitória). Molecular monitoring of the predominant genomic variants of SARS-CoV-2 circulating in Viana city was performed by Fundação Oswaldo Cruz (FIOCRUZ), Rio de Janeiro, Brazil.

#### 2.6.5 Confirmation of SARS-CoV-2 infection *via* RT-PCR of clinical specimens

Vials containing nasopharyngeal samples in viral transport media (VTM) were collected in suspected cases, until 10 days after their symptoms began. All samples were processed in a B2 Biosafety Cabinet Class II and further processed for automated viral nucleic acid extraction with the Maelstrom 4800 Nucleic Acid Extraction System (Taiwan Advanced Nanotech Inc., Taoyuan City, Taiwan) TANBead Nucleic Acid Extraction Kit (Taiwan Advanced Nanotech) according to the manufacturer’s protocol.

SARS-CoV-2 presence was confirmed using the following RT-PCR kits: 1) SARS-CoV-2 Fluorescent PCR (MACCURA, China, Target Genes: E, ORF, and N); 2) OneStep/COVID-19 (Instituto de Biologia Molecular do Paraná – IBMP, Brazil, Target Genes: ORF and N); 3) TaqPath™ COVID 19 CE IVD RT PCR Kit (Thermo Fisher Scientific, USA, Target Genes: S, ORF, and N), and 4) 2019-nCoV RUO Kit (IDT - Integrated DNA Technologies, USA, Target Genes: N1 and N2). None of the manufacturers listed above was involved in the assessment and interpretation of the study results. The reaction mixture was prepared according to the manufacturer’s instructions. Thermal cycling for either reverse transcription or amplification was performed using QuantStudio 5 and QuantStudio Real-Time PCR systems (Thermo Fisher Scientific, Waltham, MA, USA). The cut-off threshold (C_t_ value) for each sample was recorded, and samples with C_t_ values < 36 were considered positive.

### 2.7 Outcomes

#### 2.7.1 Effectiveness outcomes

The primary endpoints were the incidence rate of new cases/1,000 person-year at 90 days after 14 days of the second dose, confirmed by RT-PCR (registered at the National Laboratory Management System databank - GAL), and new cases registered at SUS National Health Surveillance Database (e-SUS VS).

The secondary outcomes were adverse events, hospitalizations and deaths. Data on hospitalization associated with SARS-CoV-2 were obtained from the Control Center of the State of Espírito Santo of assignment of public hospital beds, and the hospitalization databank (AIH) paid by the SUS. Death registration was obtained from the e-SUS VS system, Mortality Information System (SIM), and Epimed databank.

#### 2.7.2 Immunogenicity outcomes

##### 2.7.2.1 SARS-CoV-2 IgG-S antibody detection

The titers of IgG antibodies to SARS-CoV-2 spike RBD were determined using a chemiluminescent microparticle immunoassay (SARS-CoV-2 IgG II Quant Assay; Abbott Laboratories, IL, USA) according to the manufacturer’s instructions, by ARCHITECT i1000SR immunoassay analyzer (Abbott). The results are expressed in arbitrary units/mL (AU/mL). The seropositivity was defined for titers ≥ 50 AU/mL.

According to the WHO standard preparation for SARS-CoV-2 binding antibodies (11, 12), a conversion factor from Abbott AU became available (1 BAU/mL = 0.142 × AU/mL), and the results of the present study have been expressed in BAU/mL. The cut-off of 7.1 BAU/mL was used to define seropositivity.

##### 2.7.2.2 Plaque reduction neutralization test (PRNT-SARS-CoV-2 assay)

The PRNT-SARS-CoV-2 assay was carried out in 24-well tissue culture plates in a Biosafety Level 3 platform of Oswaldo Cruz Institute/FIOCRUZ (Rio de Janeiro, Brazil). Serum samples were serially diluted (1:10 to 1:31,250) and incubated with SARS-CoV-2 virus (Wuhan strain, approximately 60 plaque-forming units) at 37°C for 1 h in a humidified CO_2_ incubator. After incubation, the mixture was transferred to a 24-well tissue culture plate containing a monolayer of Vero cells (200,000 cells/well, CCL81; ATCC, Manassas, VA, USA) prepared 1 day before the test. Following incubation at 37°C for 1 h in a humidified CO_2_ incubator, the supernatant was removed, and a semi-solid medium (Medium 199 with 1.5% carboxymethylcellulose) was added. The plates were incubated for 3 days at 37°C in a humidified CO_2_ incubator, fixed in formalin, and stained with crystal violet dye. The PRNT_50_ is expressed as the reciprocal of the serum dilution able to neutralize the viral infection by 50%. Seropositivity rates were determined considering a serum dilution higher than 1:10 as the cut-off criterion for PRNT positivity.

##### 2.7.2.3 Quantification of plasma soluble mediators

The analyses of chemokines (CCL11, CXCL8, CCL3, CCL4, CCL2, CCL5, and CXCL10), pro-inflammatory cytokines (IL-1β, IL-6, TNF-α, IL-12, IFN-γ, IL-15, and IL-17), regulatory cytokines (IL-1Ra, IL-4, IL-5, IL-9, IL-10, and IL-13), and growth factors (FGF-basic, VEGF, PDGF, G-CSF, GM-CSF, IL-7, and IL-2) were carried out with the Luminex Platform (Bio-Plex Pro™ Human Cytokine 27-Plex Assay; Bio-Rad Laboratories, Hercules, CA, USA). Acquisition was accomplished with the Luminex 200 System, and data were analyzed using Manager software. The results are expressed as pg/mL, estimated by five-parameter logistic regression according to the standard curve.

### 2.8 Statistical analyses

#### 2.8.1 Statistical analyses of effectiveness

The non-inferiority analysis of effectiveness followed a mixed-effects Poisson model. The number of confirmed cases was the outcome, which was obtained as counts of cases in the surveillance database. Age group was used as the covariate. The number of cases, aggregated over a subset *i* given by age group and vaccination status was given by a Poisson distribution, y_i_ ~ Poisson(λ_i_), where λ_i_ is the link variable. The formula for this link variable uses the age group f, the vaccination status v, and the total person-time component T_i_ in the group by: log(λ_i_) = log(T_i_) + βv + δ_i_, Where δ_i_ and β are random effects. In particular, the criterion for comparing half dose and full dose for confirmed cases is whether there is a significant difference for the β parameter evaluated between the ChAd Half Dose and ChAd Full Dose groups. The total person-time is calculated as the product of population size, stratified by age/sex, and time minus the sum of all individual times, counting all vaccines, after first dose of vaccination and 14 days. Statistical evaluation using information confirmed by RT-PCR provides more specificity, in which case the model is modified to have y_i_ ~ Poisson (λ_i_), where λ_i_ is given by T_i_ in the group by: log(λ_i_) = log(T_i_) + γs + δ_i_, using by T_i_ as the person-time component and s indicates the dose group (ChAd Half Dose/ChAd Full Dose). Parameter γ permits comparison of non-inferiority between the groups (group coefficient criterion). The estimate after Monte Carlo Markov Chain (MCMC) simulations provides an estimate of the parameter gamma. The 95% credibility interval (CrI) was used to signal significant statistical differences between the ChAd Half Dose and ChAd Full Dose groups, when the lower bound of the interval was positive or the upper bound of the interval was negative. The analysis was done with the R platform (version 4.0.4) and JAGS (package R2jags) as Bayesian analysis tool for performing MCMC analysis (12,000 iterations; 2,000 burn-in; 3 chains; parameter thin=2). The methods were based on Bayesian models and estimates and 95% credibility intervals given by the intervals between 2.5 and 97.5 percentiles. Priors for the random effects δ_i,_ γ and β were normal distributions with zero mean and precision 0.01. Additional comparative analyses between groups were performed by Mann-Whitney test. Chi-square test was employed to compare categorical data. A limit of p ≤ 0.05 was considered statistically significant.

New cases were calculated based on confirmed RT-PCR (registered at GAL databank) or new cases registered at e-SUS VS databank. The numbers of deaths and hospitalizations were compared between the ChAd Full Dose and ChAd Half Dose groups. Record linkage was carried out to integrate all databases from the health information systems, including vaccine status (www.vianavacinada.sesa.es.gov.br and www.vacinaeconfia.sesa.es.gov.br), new cases of SARS-Cov-2 infection (GAL and e-SUS VS), hospitalization (Bed Regulation Center of the Health Department of the State of Espírito Santo and AIH databank), deaths (e-SUS VS, SIM and Epimed databank), and AEs (*e-SUS Notifica* and www.vianavacinada.sesa.es.gob.br). Cases are linked to the vaccination records with a linkage tool (13), using matching information.

#### 2.8.2 Statistical analyses of immunogenicity parameters

The subsample size for the immunogenicity assays comprised 558 participants, stratified by age (18–29, 30–39, 40–49 years old) calculated by the WINPEPI System (14), with a percent accuracy of 10 points, differences in geometric means of antibody titers of 100% (ratio > 2), study power of 90%, and alpha error of 5%.

Data analysis was performed using GraphPad Prism v.9.1.1 software (San Diego, CA, USA). Data normality distribution was assessed by the Shapiro-Wilk test. Considering the non-parametric distribution of the data sets, multiple comparative analyses of serological data and soluble mediators among subgroups were carried out by the Kruskal-Wallis test, followed by Dunn’s post-test. Comparative analyses between two subgroups were performed by the Mann-Whitney test. Chi-square test was employed to compare categorical data. Correlation between the SARS-CoV-2 IgG II Quant assay and PRNT was assessed with Spearman’s coefficient, whereas agreement was assessed with the Kappa index. P ≤ 0.05 was considered statistically significant.

Signatures of plasma soluble mediators were constructed by first converting the original data expressed as baseline fold into categorical data using the global median values to estimate the proportion of subjects above the cut-off edge. Thereafter, the phenotypes and functional features reaching the proportion of subjects higher than 50% were considered for further analyses.

Cytoscape software platform (available at http://cytoscape.org) was employed to construct networks of plasma soluble mediators based on Spearman’s correlation “r” scores. Networks were assembled with clustered layout, with nodes representing each plasma soluble mediator. Connecting edges illustrate correlations between pairs of attributes. Correlation matrices were generated using R software (Project for Statistical Computing Version 3.0.1).

## 3 Results

### 3.1 Effectiveness and safety

**Figure 2:**
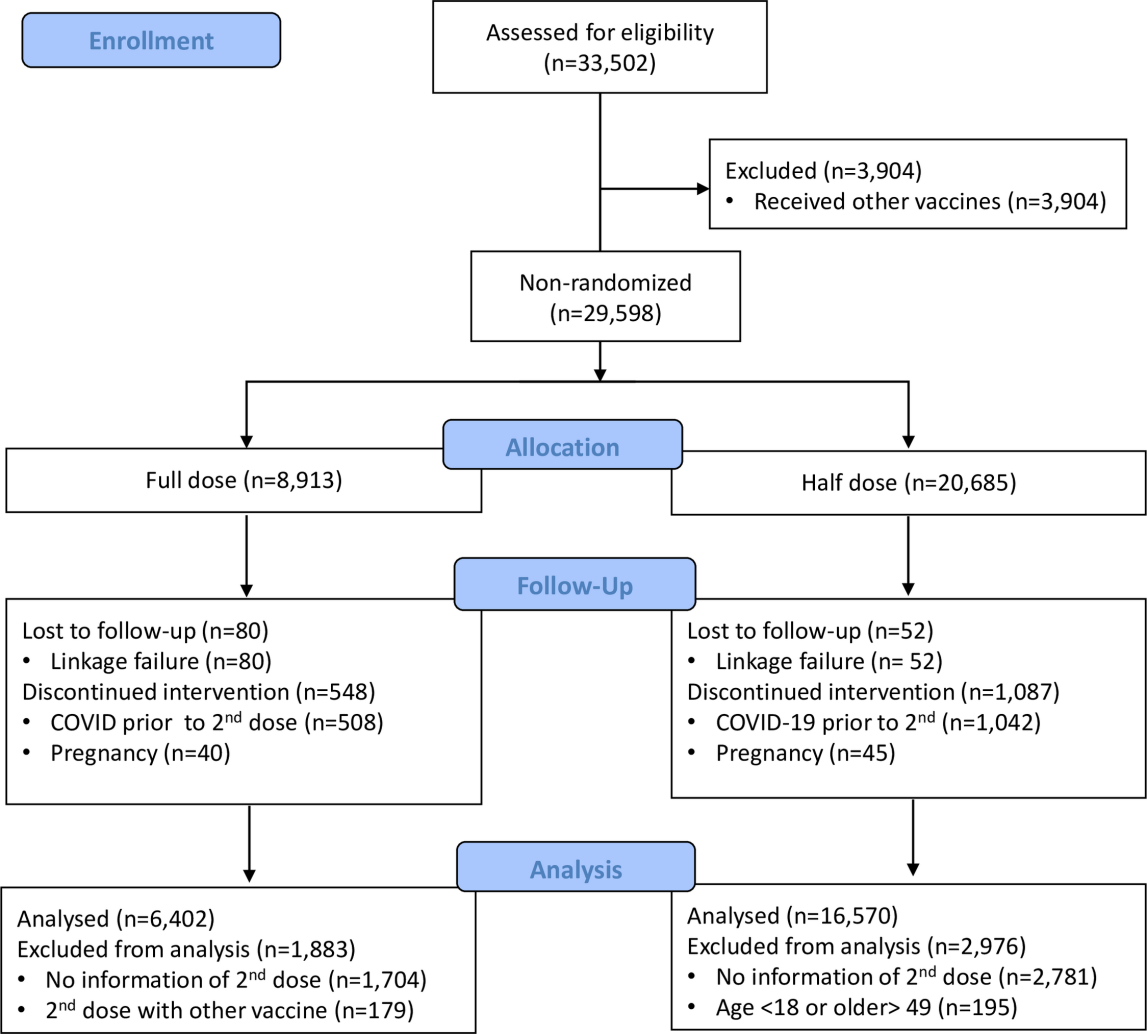
CONSORT flow diagram.

A total of 29,598 individuals were included in the study population: 20,685 subjects received half dose (ChAd Half Dose) and 8,913 received full dose (ChAd Full Dose). In the ChAd Half Dose group, 195 were excluded because they were not 18 to 49 years of age, 2,781 received only the first dose, 1,042 had COVID prior to the second dose, 45 got pregnant, and another 52 had failure for linkage of databanks. In the ChAd Full Dose group, 1,704 received only the first dose and were excluded, 508 had COVID prior to the second dose, 40 got pregnant, and 80 were excluded because of databank linkage error. Finally, 16,570 and 6,402 were included for analyses in the ChAd Half Dose and ChAd Full Dose groups, respectively (**Figure 2**). The ChAd Full Dose group was slightly older and had more females (**Table 1**).

**Table 1:** Demographic characteristics of the study population.

Groups	Full dose (n = 6,402)								Half dose (n = 16,570)							
Study groups by Age	Female	%	Male	%	Missing	%	Total	%	Female	%	Male	%	Missing	%	Total	%
18–29	799	55	587	41	57	4	1,443	22	3,360	49	3,333	49	143	2	6,836	41
30–39	1,172	54	902	42	79	4	2,153	34	2,637	46	2,952	52	137	2	5,726	35
40–49	1,572	56	1,180	42	54	2	2,806	44	1,752	44	2,190	55	66	2	4,008	24
Total	3,543	55	2,669	42	190	3	6,402	100	7,749	47	8,475	51*	346	2	16,570	100
Mean Age	36.8		36.8		34.0		36.7		31.9*		32.8*		31.8		32.3*	
Age SD	8.6		8.6		7.9		8.6		8.6		8.6		7.8		8.6	

SD, standard deviation; Full dose, ChAdOx1 Full dose group; Half dose, ChAdOx1 Half dose group. *p < 0.05.

This is a populational study. We have planned to include 70% of the population.

The population of Viana aged 18-49 is 33,502 inhabitants. We have included 29,598 participants (88% of the inhabitants). After exclusions, the study included 22,972 participants in the analysis (68.6% of the entire city population).

Under the surveillance system (e-SUS VS), an incidence of 44.4 cases per 1,000 person-year was estimated for the ChAd Half Dose group, which was close to the observed incidence in the ChAd Full Dose group of 49.8 cases per 1,000 persons-year **Table 2**.

**Table 2:** Effectiveness of Full and Half dose of ChAdOx1 nCoV-19 in the Viana population.

Parameters	Full dose (n = 6,402)			Half dose (n = 16,570)		
All Cases						
Age Groups	Cases/person-day	Incidence (per 1,000 person-year)	1-RR (%)	Cases/person-day	Incidence (per 1,000 person-year)	1-RR (%)
18–29	16/100,379	58.2	48.3	72/597,962	43.9	61.0
30–39	21/159,195	48.1	67.0	61/501,607	44.4	69.6
40–49	27/209,403	47.1	74.9	43/348,504	45.0	76.0
All	64/468,977	49.8	65.1	176/1,448,073	44.4	68.9
	Cases Confirmed by RT-PCR					
Age Groups	Cases/person-day	Incidence (per 1,000 person-year)		Cases/person-day	Incidence (per 1,000 person-year)	
18–29	6/100,379	21.8		43/597,962	26.2	
30–39	11/159,195	25.2		31/501,607	22.6	
40–49	16/209,403	27.9		20/348,504	20.9	
All	33/468,977	25.7		94/1,448,073	23.7	
Model-based Analysis Group Coefficient	- 0.09 (-0.49 to 0.31)					

CrI, Credibility Interval; Full dose, ChAdOx1 Full dose group; Half dose, ChAdOx1 Half dose group.

After complete immunization (2 doses), the number of new cases confirmed by RT-PCR in the ChAd Half Dose group was 94 cases per 1,448,073 persons-day, resulting in 23.7 cases per 1,000 persons-year. In the ChAd Full Dose group, 33 cases per 468,977 persons-day were confirmed by RT-PCR, and incidence was evaluated at 25.7 cases per 1,000 persons-year. The range from -0.49 to 0.3 for the coefficient of the group comparison model indicated no statistical difference between the incidence of both groups (**Table 2**). After adjusting for age and sex, there was no difference between the ChAd Full Dose and ChAd Half Dose groups (**Table 3**). The overlapping intervals and the group coefficient signal that the effectiveness of the half dose group was non-inferior the one for ChAd Full Dose group, for overall confirmed cases and confirmed by RT-PCR.

**Table 3:** Effectiveness of Full and Half dose of ChAdOx1 nCoV-19 in the Viana population after adjusting for age and sex.

Parameters	Full dose (n=6,402)			Half dose (n=16,570)		

All Cases
Age Groups	Cases/person-day	Incidence (per 1,000 person-year)	1-RR (%)	Cases/person-day	Incidence (per 1,000 person-year)	1-RR (%)
Males						
18–29	9/38,993	84.2	14.9	36/289,341	45.4	54.1
30–39	7/63,240	40.4	64.4	27/257,544	38.3	66.2
40–49	9/83,691	39.3	76.1	23/190,010	44.2	73.1
All	25/185,924	49.1	59.0	86/736,895	42.6	64.4
Females						
18–29	7/59,916	42.6	65.5	36/296,591	44.3	64.1
30–39	14/93,381	54.7	69.9	34/232,206	53.4	70.6
40–49	18/124,078	53.0	74.2	20/153,036	47.7	76.7
All	39/277,375	51.3	68.8	90/681,833	48.2	70.7
Cases Confirmed by RT-PCR						
Age Groups	Cases/ person-day	Incidence (per 1,000 person-year)		Cases/ person-day	Incidence (per 1,000 person-year)	
Males						
18–29	3/38,993	28.1		18/289,341	22.7	
30–39	3/63,240	17.3		14/257,544	19.8	
40–49	3/83,691	13.1		11/190,010	21.1	
All	9/185,924	17.7		43/736,895	21.3	
Females						
18–29	3/59,916	18.3		25/296,591	30.7	
30–39	8/93,381	31.3		17/232,206	26.7	
40–49	13/124,078	38.2		9/153,036	21.5	
All	24/277,375	31.6		51/681,833	27.3	
Model-based Analysis Group oefficient	-0.04 (-0.44 to 0.37)					

CrI, Credibility Interval; Full dose, ChAdOx1 Full dose group; Half dose, ChAdOx1 Half dose group.

The frequency of AEs was similar in both groups (**Table 4**), but the duration of symptoms was shorter in ChAd Half Dose group, especially after first dose. No serious AEs, hospitalizations, or deaths were reported in either group. Overall rate of adverse events was 80.5% and 50.8% after first and second dose (**Tables 4**, **5**). Reactogenicity was lower after the second dose in both the ChAd Half Dose and ChAd Full Dose groups (**Tables 4**, **5**).

**Table 4:** Adverse events frequency and duration after first and second Full and Half Dose of ChAdOx1 nCov-19.

Adverse Event (%)	Full dose (n = 154)			Half dose (n = 558)		
	1^st^ dose (%)	2^nd^ dose (%)	Duration (days)	1^st^ dose (%)	2^nd^ dose (%)	Duration (days)
Overall	84	57	–	83	52	–
Pain on injection local	77	59	4.5	69*	34*	3.1**
Headache	51	37	3.7	50	21*	2.5*
Malaise	–	–	–	47	25	2.3*
Tiredness	43	28	4.6	41	16	2.9
Chills	–	–	–	37	18	1.9
Muscle pain	35	24	9.3	36	14	2.9**
Joint pain	19	7	14.1	30*	12*	2.9**
Fever	17	14	1.7	23	8	1.7
Nausea	17	6	3.9	17	8	2.0
Local hardening	–	10	3.5	31	16*	3.1
Local edema	14	17	4.6	24	12	2.5
Redness	10	11	2.4	13	8*	3.0
Vomiting	1	0	1.0	2	1	2.4

Full dose, ChAdOx1 Full dose group; Half dose, ChAdOx1 Half dose group. *p < = 0.05 **p < = 0.001.

**Table 5:** Local and systemic adverse events after Half dose in Viana population.

Adverse Events	1^st^ dose (n = 6,102)	2^nd^ dose (n = 827)	1^st^ dose (%)	2^nd^ dose (%)
Overall	4,913	420	80.5	50.8
Local symptoms	3,163	269	51.8	32.5
Redness	778	87	12.8	10.5
Local edema	2,137	177	35.0	21.4
Local hardening	2,092	171	34.0	20.7
Hematoma	365	41	5.9	4.9
Local heat	1,916	160	31.4	19.4
Systemic symptoms	4,510	315	73.9	38.1
Itching	706	60	11.6	7.3
Fever	1,865	67	30.6	8.1
Chill	2,960	115	48.5	13.9
Nausea/vomiting	935	42	15.3	5.1
Malaise	3,344	163	54.8	19.7
Headache	3,560	200	58.3	24.2
Joint pain	2,108	86	34.6	10.4
Muscle pain	3,016	156	49.4	18.9
Fatigue (tiredness)	1,983	107	32.5	12.9

Half dose, ChAdOx1 Half dose group.

From all included 20.685 individuals, 6,102 brought back diaries after first dose and 827 after second dose.

### 3.2 Humoral reactivity

**Figure 3:**
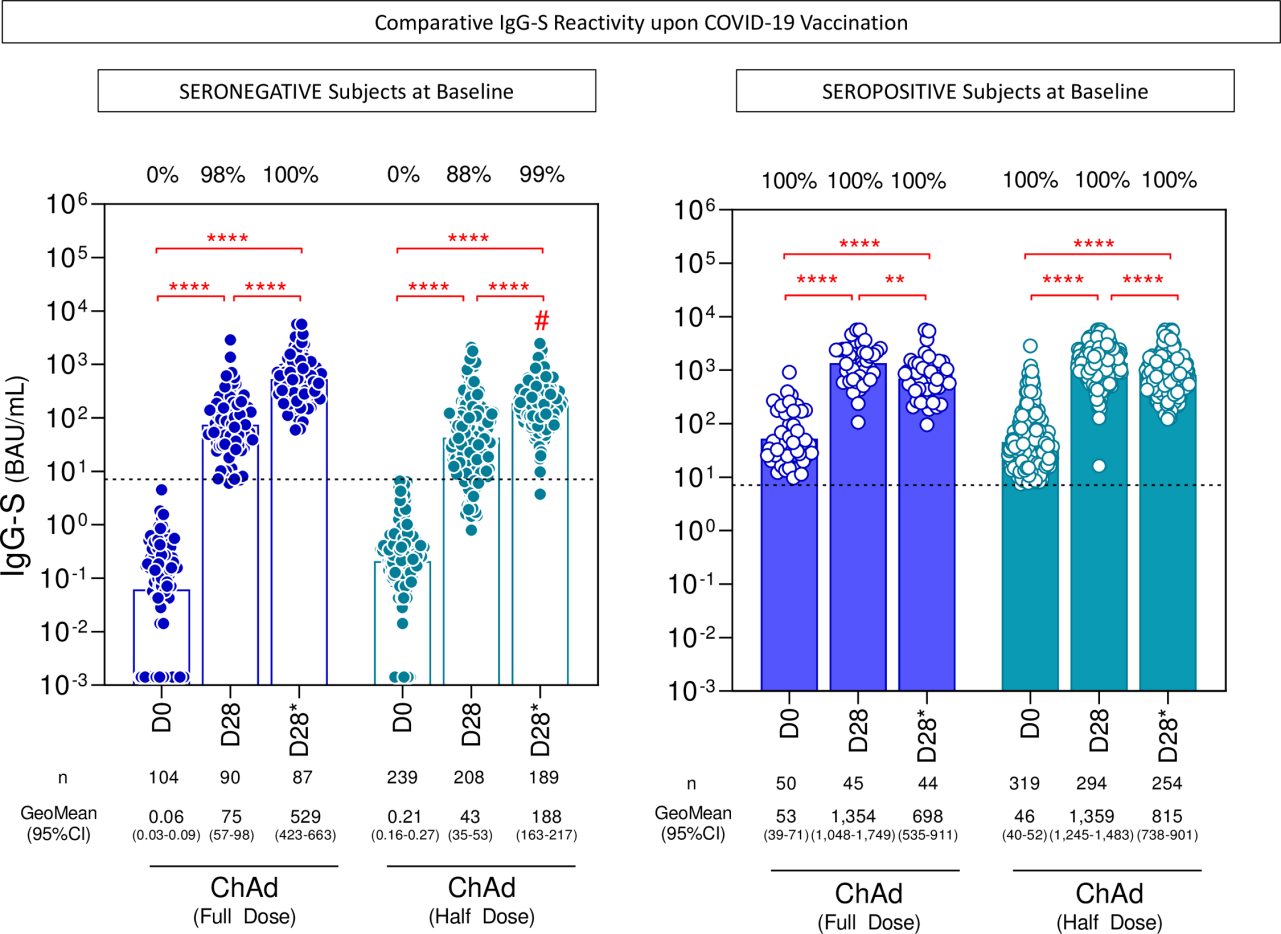
SARS-CoV-2 IgG-S reactivity upon COVID-19 vaccination. The titers of IgG antibodies to SARS-CoV-2 spike receptor-binding domain (IgG-S) were analyzed in serum samples from subjects with seronegative and seropositive status at baseline at consecutive timepoints: prior (D0), after first (D28) and second (D28*) doses, including subjects receiving a half dose (ChAd Half Dose, n = 239, 208, 189 and 319, 294, 254, respectively), represented by light blue symbols on the right side of each graph, compared to reference volunteers receiving the standard dose (ChAd Full Dose, n = 104, 90, 87 and 50, 45, 44, respectively), represented by dark blue symbols on the left side of each graph. The levels of IgG-S were determined by the chemiluminescent microparticle immunoassay as described in the Methods. The seropositivity was defined for titers ≥ 7.1 BAU/mL (dashed line). Data are presented as a scatter plot of IgG-S titers at D0, D28, and D28* over bars representing the geometric mean (GeoMean) titers. The chi-square test was employed for comparative analyses of IgG-S seropositivity rates among groups. Multiple comparisons of IgG-S titers among subgroups were carried out by Kruskal-Wallis test followed by Dunn’s post-test for sequential pairwise comparisons. Significant differences were considered at p ≤ 0.05 (* represents the p value power; **p≤0.01, **** p≤0.0001) and are indicated by connecting lines and # symbol for intragroup (D0 vs. D28 vs. D28*) and intergroup ChAd Full Dose versus ChAd Half Dose comparisons, respectively.

**Figure 4:**
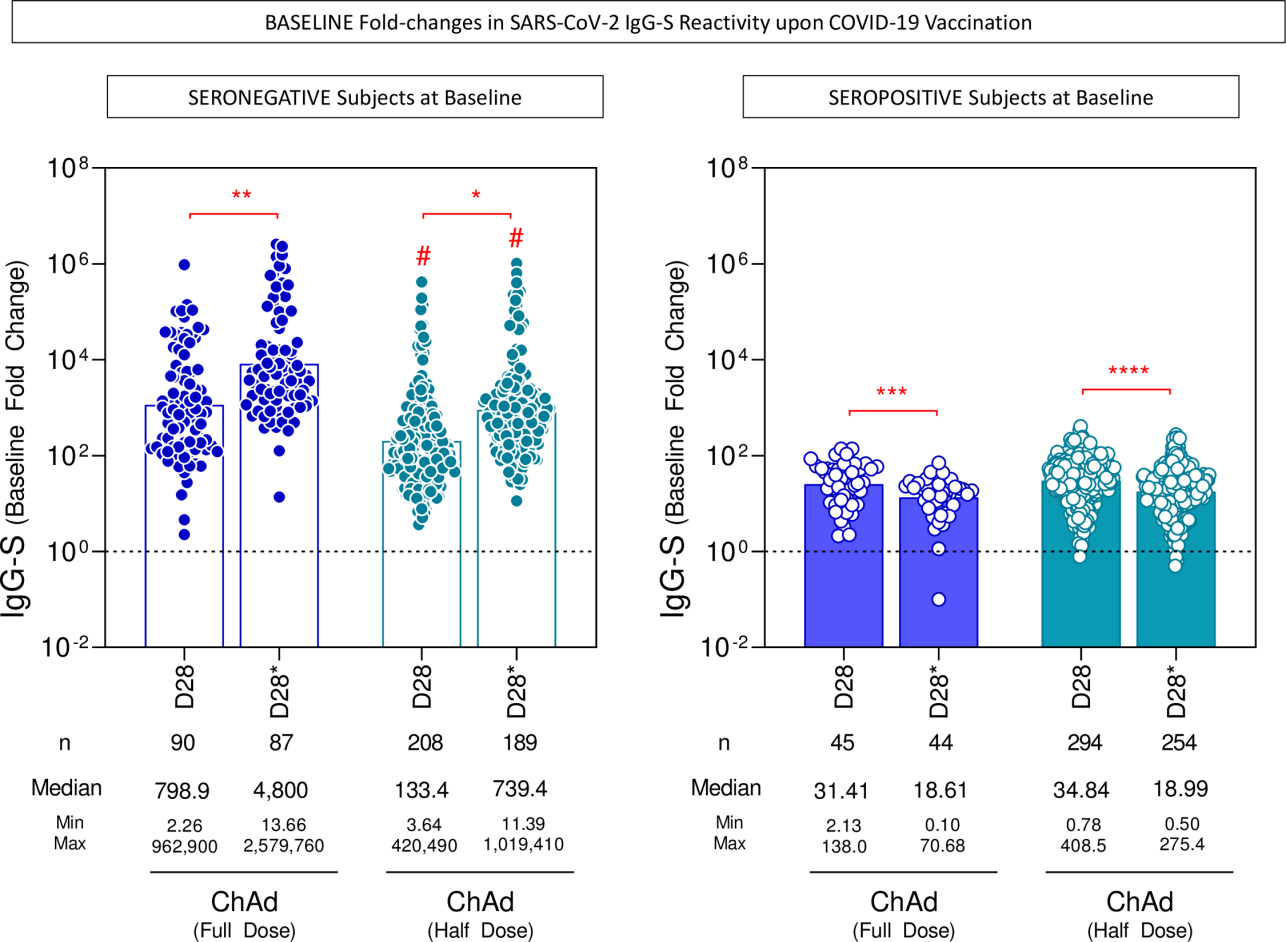
Baseline fold changes in SARS-CoV-2 IgG-S reactivity upon COVID-19 vaccination. The baseline fold changes of IgG antibody titers to SARS-CoV-2 spike receptor-binding domain (IgG-S) were analyzed in serum samples from subjects with seronegative and seropositive status at baseline at distinct timepoints: after first (D28) and second (D28*) doses, including subjects receiving a half dose (ChAd Half Dose), n = 208, 189 and 294, 254, respectively), represented by light blue symbols on the right side of each graph, as compared to the reference volunteers receiving the standard-dose (ChAd Full Dose), n = 90, 87 and 45, 44, respectively), represented by dark blue symbols on the left side of each graph in relation to the levels observed prior vaccination (D0). The levels of IgG-S were determined by the chemiluminescent microparticle immunoassay and the baseline fold changes were calculated as described in the Methods. The changes in IgG-S reactivity were assessed defining the fold-change = 1 as the reference for unaltered levels (dashed line). Data are presented as a scatter plot of baseline fold-changes in IgG-S titers at D28 and D28* according to the individual baseline values observed at D0 (D28/D0 and D28*/D0) over bars representing the median fold changes. Comparative analyses of baseline fold changes in IgG-S titers were performed by Mann-Whitney test. Significant differences were considered at p ≤ 0.05 (* represents the p value power; * p≤0.05, **p≤0.01, *** p≤0.001, **** p≤0.0001) and are indicated by connecting lines and # symbol for intragroup (D28 vs D28*) and intergroup ChAd Full Dose vs ChAd Half Dose comparisons, respectively.

The anti-SARS-CoV-2 spike (S) protein receptor binding domain (RBD) (anti-IgG-S) was analyzed by chemiluminescence and the neutralizing antibodies by plaque reduction neutralization test (PRNT), in a subsample of 558 participants who received two half doses and an external comparison group (154 healthcare workers) who received two full doses. The ChAd Full Dose group was slightly older and had more females (**Table 6**). After the second dose, the seroconversion rate of IgG-S in seronegative individuals at baseline (n =239) in the ChAd Half Dose was 99.5%, similar to that in the ChAd Full Dose of 100% (p > 0.9999). The geometric mean concentration (GeoMean) of anti-IgG-S (95% CI; BAU/mL) were ChAd Half Dose = 188 (163–217) and ChAd Full Dose = 529 (423-663) BAU/mL, (p ≤ 0.001), 28 days after the second dose (**Figure 3**). In the subgroup of subjects with seropositive status at baseline (pre-immune individuals), the first dose induced very high and similar IgG-S GeoMean concentration (ChAd Half Dose = 95% CI: 1,359 [1,245-1,483] and ChAd Full Dose = 95% CI: 1,354 [1,048–1,749]) (**Figure 3**), and was able to induce a 35-fold increase in baseline IgG-S reactivity similar to one full dose (**Figure 4**). In these pre-immune individuals, the second dose decreased the antibody levels in both groups (ChAd Half Dose = 95% CI: 815 [738-901] and ChAd Full Dose = 95% CI: 698 [535-911]) BAU/mL (**Figure 3**).

**Table 6:** Demographic characteristics of the immunogenicity study subsample.

	Full dose (n = 154)	Half dose (n = 558)
Mean Age ± SD	39.2 ± 6.4	33.3 ± 8.5*
Female %	84	50*

SD, standard deviation; Full dose, ChAdOx1 Full dose group; Half dose, ChAdOx1 Half dose group. *p < 0.05.

**Figure 5:**
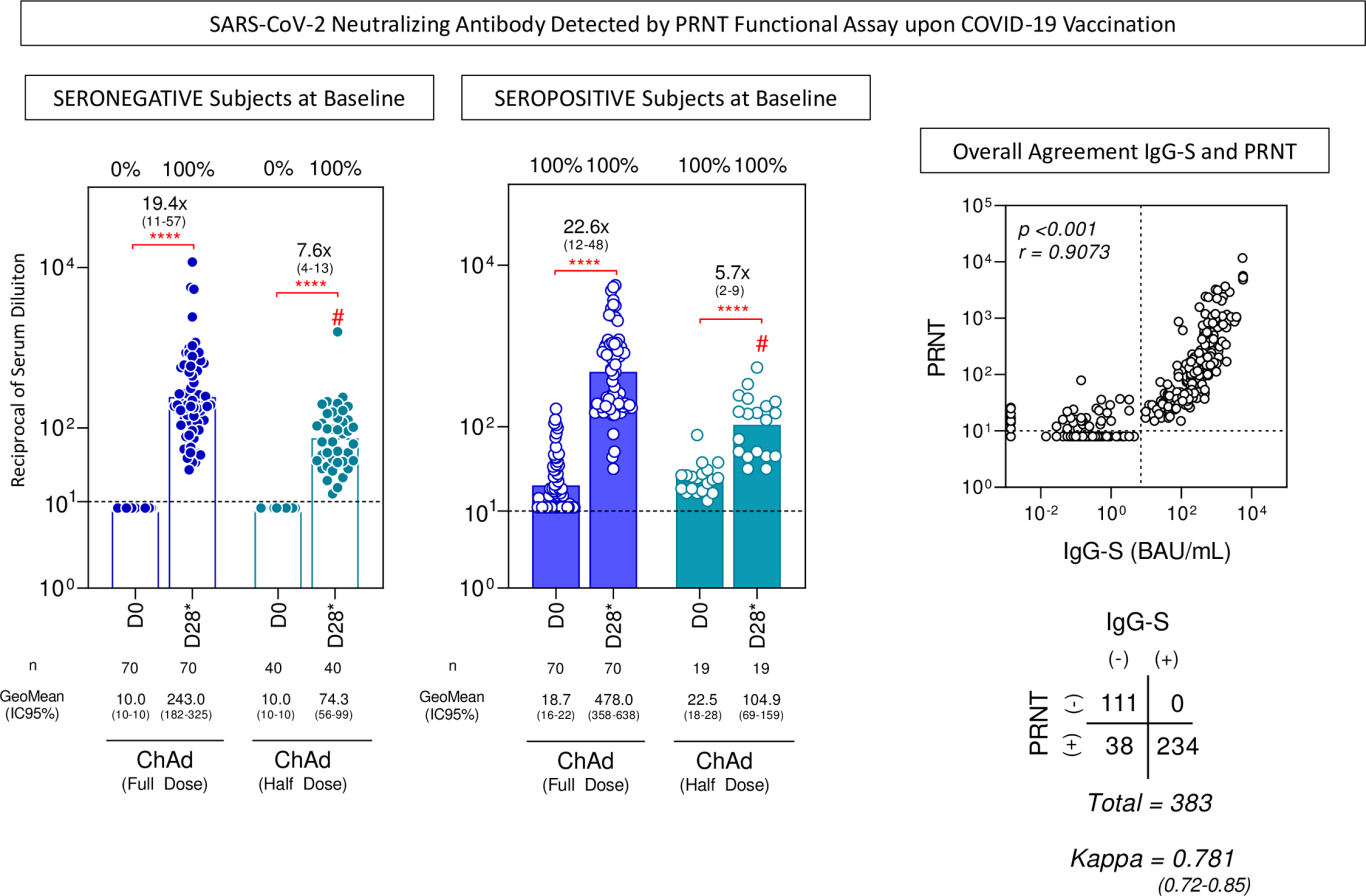
SARS-CoV-2 neutralizing antibody detected by the PRNT functional assay upon COVID-19 vaccination. The levels of SARS-CoV-2-specific neutralizing antibodies were detected by the PRNT in serum samples from subjects with seronegative and seropositive status at baseline at distinct timepoints: after first (D28) and second (D28*) doses, including subjects receiving a half dose (ChAd Half Dose), n = 40 and 19, respectively), represented by light blue symbols on the right side of each graph compared to the reference volunteers receiving the standard dose (ChAd Full Dose), n = 70 and 70, respectively), represented by dark blue symbols on the left side of each graph. Seropositivity rates were determined considering the serum dilution higher that 1:10 as the cut-off criterion for PRNT positivity (dashed line). The data are presented as a scatter plot of PRNT titers at D0 and D28*, expressed as reciprocal of serum dilution over bars representing the geometric mean (GeoMean)titer. Chi-square test was employed for comparative analysis of PRNT seropositivity rates among groups. Comparative analysis of PRNT titers was performed by Mann-Whitney test. Significant differences were considered at p ≤ 0.05(* represents the p value power; **** p≤0.0001) and are indicated by connecting lines and # symbol for intragroup (D0 vs D28*) and intergroup ChAd Full Dose versus ChAd Half Dose comparisons, respectively. Spearman’s correlation test and Kappa index were used to assess the overall agreement between serological tests. The results are presented as a scatter distribution for paired samples and the scores are provided in the figure.

Similarly, the rate of seroconversion of neutralizing antibodies by PRNT was 100% in seronegative subjects at baseline for both vaccination regimens (ChAd Half Dose = 95% CI: 74.3 [56–99] and ChAd Full Dose = 95% CI: 243.0 [182–325]) (**Figure 5**). GeoMean concentration of neutralizing antibodies was very high in pre-immune individuals in both groups (ChAd Half Dose = 95% CI: 104.9 [69–159] and ChAd Full Dose = 95% CI: 478.0 [358–638]). The agreement between IgG-S titers detected by the chemiluminescence assay and neutralizing antibodies detected by the PRNT was moderate to high (kappa = 0.781 [0.72–0.85]) with a high correlation score (r = 0.9073, p < 0.001) (**Figure 5**).

### 3.3 Overall profile of plasma soluble mediators upon COVID-19 vaccination

**Figure 6:**
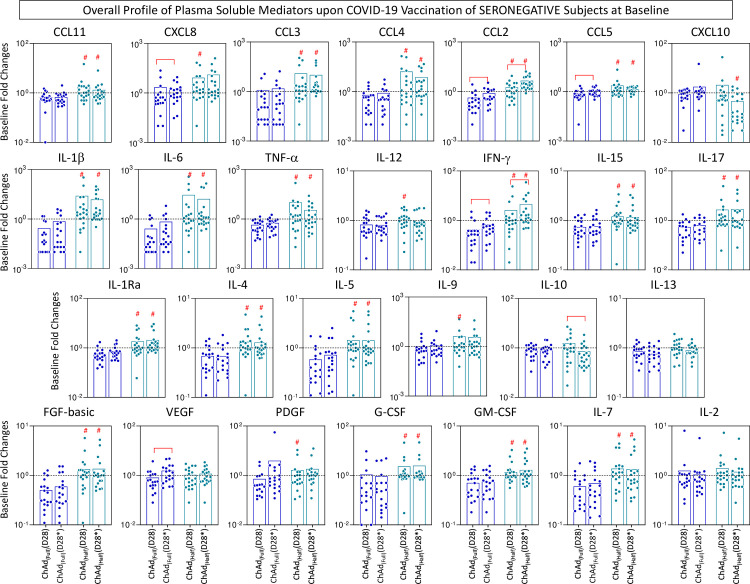
Overall profile of plasma soluble mediators upon COVID-19 vaccination of seronegative subjects at baseline. The levels of chemokines (CCL11, CXCL8, CCL3, CCL4, CCL2, CCL5, CXCL10), pro-inflammatory cytokines (IL-1β, IL-6, TNF-α, IL-12, IFN-γ, IL-15, IL-17), regulatory cytokines (IL-1Ra, IL-4, IL-5, IL-9, IL-10, IL-13), and growth factors (FGF-basic, VEGF, PDGF, G-CSF, GM-CSF, IL-7 and IL-2) were measured in plasma samples from subjects with seronegative status at baseline in consecutive timepoints: prior (D0), after first (D28) and second (D28*) doses, including subjects receiving a half dose (ChAd Half Dose), n = 20), represented by light blue symbols on the right side of each graph compared to the reference volunteers receiving the standard dose (ChAd Full Dose), n = 18), represented by dark blue symbols on the left side of each graph. Quantification of plasma soluble mediators was carried by the high-throughput Luminex Microbeads Platform as described in the Methods. The results are presented as white bar charts of median values expressed in fold changes in concentrations (pg/mL) according to the individual baseline values (D28/D0 and D28*/D0), referred as ChAd Full (D28), ChAd Full (D28*), ChAd Half (D28) and ChAd Half (D28*). Significant differences were considered at p ≤ 0.05 and are indicated by connecting lines and # symbol for intragroup (D28 vs. D28*) and intergroup ChAd Full Dose versus ChAd Half Dose comparisons, respectively.

**Figure 7:**
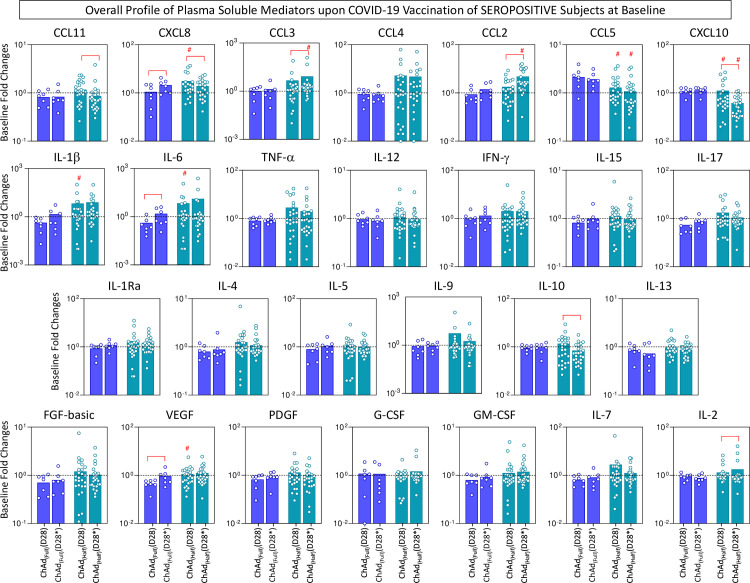
Overall profile of plasma soluble mediators upon COVID-19 vaccination of seropositive subjects at baseline. The levels of chemokines (CCL11, CXCL8, CCL3, CCL4, CCL2, CCL5, CXCL10), pro-inflammatory cytokines (IL-1β, IL-6, TNF-α, IL-12, IFN-γ, IL-15, IL-17), regulatory cytokines (IL-1Ra, IL-4, IL-5, IL-9, IL-10, IL-13), and growth factors (FGF-basic, VEGF, PDGF, G-CSF, GM-CSF, IL-7 and IL-2) were measured in plasma samples from subjects with seropositive status at baseline in consecutive timepoints: prior (D0), after first (D28) and second (D28*) doses, including subjects receiving a half dose (ChAd Half Dose), n = 26), represented by light blue symbols on the right side of each graph compared to the reference volunteers receiving the standard dose (ChAd Full Dose), n = 7), represented by dark blue symbols on the left side of each graph. Quantification of plasma soluble mediators were carried by high-throughput Luminex Microbeads Platform as described in the Methods. The results are presented as color filled bar charts of median values expressed in fold changes in plasma concentrations (pg/mL) according to the individual baseline values (D28/D0 and D28*/D0), referred as ChAd Full (D28), ChAd Full (D28*), ChAd Half (D28) and ChAd Half (D28*). Significant differences were considered at p ≤ 0.05 and are indicated by connecting lines and # symbol for intragroup (D28 vs. D28*) and intergroup ChAd Full Dose versus ChAd Half Dose comparisons, respectively.

The overall profile of plasma chemokines (CCL11, CXCL8, CCL3, CCL4, CCL2, CCL5, CXCL10), pro-inflammatory cytokines (IL-1β, IL-6, TNF-α, IL-12, IFN-γ, IL-15, IL-17), regulatory cytokines (IL-1Ra, IL-4, IL-5, IL-9, IL-10, IL-13), and growth factors (FGF-basic, VEGF, PDGF, G-CSF, GM-CSF, IL-7, and IL-2) was characterized on D28 and D28* upon COVID-19 vaccination with a half dose compared to the full dose. The results are presented in seronegative and seropositive subjects at baseline (**Figures 6**, **7**). Data analyses were carried out considering the fold changes in plasma concentrations according to individual baseline values (D28/D0 and D28*/D0). The results reported for subjects with seronegative status at baseline demonstrated that overall, most chemokines (except CXCL10), cytokines (except IL-10 and IL-13), and growth factors (except VEGF and IL-2) were higher in the ChAd Half Dose group compared to the ChAd Full Dose group (**Figure 6**). Conversely, the results reported for subjects with seropositive status at baseline demonstrated that there were generally no significant differences between the ChAd Half Dose and ChAd Full Dose groups, except for several chemokines (CXCL8, CCL3, CCL2, CCL5, CXCL10), pro-inflammatory cytokines (IL-1 β, IL-6), and growth factors (VEGF) (**Figure 7**).

### 3.4 Panoramic overview of plasma soluble mediator signatures upon COVID-19 vaccination

**Figure 8:**
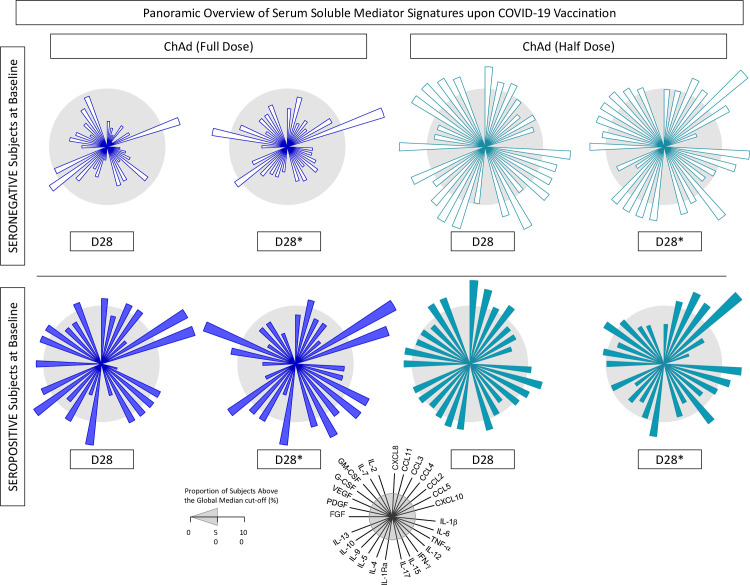
Panoramic overview of plasma soluble mediator signatures upon COVID-19 vaccination. Overall signature of chemokines (CCL11, CXCL8, CCL3, CCL4, CCL2, CCL5, CXCL10), pro-inflammatory cytokines (IL-1β, IL-6, TNF-α, IL-12, IFN-γ, IL-15, IL-17), regulatory cytokines (IL-1Ra, IL-4, IL-5, IL-9, IL-10, IL-13), and growth factors (FGF-basic, VEGF, PDGF, G-CSF, GM-CSF, IL-7, and IL-2) were collected for subjects with seronegative or seropositive status at baseline in distinct timepoints: after first (D28) and second (D28*) doses, including vaccinees receiving the half dose (ChAd Half Dose), n = 20 and 26, respectively, represented by light blue symbols on the right side of each graph, as compared to the reference volunteers receiving the standard-dose (ChAd Full Dose), n = 18 and 7, respectively, represented by dark blue symbols on the left side of each graph. Signatures of plasma soluble mediators were built as described in the Methods, by first converting the plasma levels of soluble mediators, originally expressed as baseline fold (D28/D0 and D28*/D0) into categorical data (percentual, %) using the median values of each plasma mediator as the cut-off to identify the proportion of subjects above the cut-off edges. The final data are shown in radar chart, with each axis represents one plasma mediators. The 50^th^ percentile (gray zone) was used to underscore the plasma soluble mediators with increased proportion (≥50%) in each study group.

The overall snapshot of plasma soluble mediator signatures was taken on 28 days after first (D28) and second dose (D28*) upon COVID-19 vaccination with a half dose compared to the full dose. The results are presented in **Figure 8**. Data observed for subjects with seronegative status at baseline demonstrated that, in general, the proportion of the ChAd Half Dose group presenting with higher levels of chemokines, cytokines, and growth factors was increased compared to the ChAd Full Dose group (**Figure 8**). On the other hand, plasma soluble mediator signatures of subjects with seropositive status at baseline showed similar profiles between the ChAd Half Dose and ChAd Full Dose groups, underscoring that regardless of the vaccination regimen, an increased proportion of subjects presented with higher levels of chemokines, cytokines, and growth factors (**Figure 8**).

### 3.5 Integrative correlation matrices and networks of plasma soluble mediators upon COVID-19 vaccination

**Figure 9:**
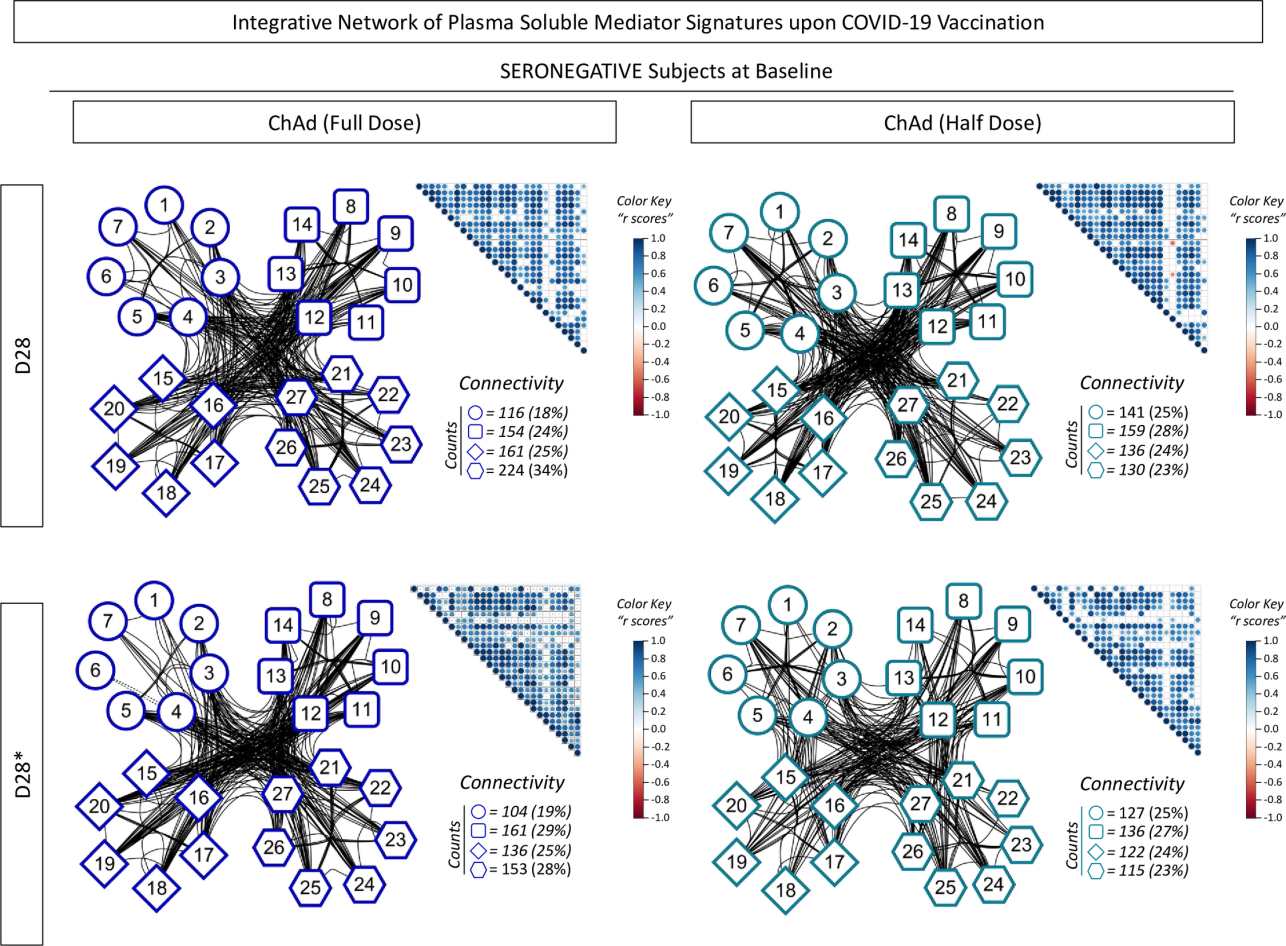
Integrative network of plasma soluble mediator signatures upon COVID-19 vaccination. Comprehensive correlation matrices were assembled based on the Spearman “r” scores between chemokines (1 to 7, representing: CCL11, CXCL8, CCL3, CCL4, CCL2, CCL5, CXCL10, respectively), pro-inflammatory cytokines (8 to 14, representing: IL-1β, IL-6, TNF-α, IL-12, IFN-γ, IL-15, IL-17, respectively), regulatory cytokines (15 to 20, representing: IL-1Ra, IL-4, IL-5, IL-9, IL-10, IL-13, respectively, and growth factors (21 to 27, representing: FGF-basic, VEGF, PDGF, G-CSF, GM-CSF, IL-7 and IL-2, respectively) measured in plasma samples from subjects with seronegative status at baseline in distinct timepoints: after first (D28) and second (D28*) doses, including subjects receiving the low dose (ChAd Half Dose, n = 20), represented by light blue symbols on the right side of each graph compared to the reference volunteers receiving the standard dose (ChAd Full Dose, n = 18). The soluble mediators were measured by high-throughput microbeads array as described in the Methods. Panoramic correlation overviews are shown as triangle template matrices with each square intersection representing the correlation “r” score between pairs of soluble mediators. The “r” scores are represented by circles of proportional sizes, scaled from -1 to +1 with gradient color key for negative (red circles) or positive (blue circles) correlations. The white squares represent non-significant correlations. Networks were built using a clustered layout, with nodes representing each plasma soluble mediators. Connecting edges illustrate correlations between pairs of attributes. Intrinsic connectivity for each immune mediator category (chemokines=○, pro-inflammatory cytokine=□, regulatory cytokine=◊, and growth factors=o) are provided in the figure.

To better understand the complex data set of interrelationships between distinct soluble mediators upon COVID-19 vaccination, integrative correlation analysis was carried out, and comprehensive matrices were created to assemble networks to assess the neighborhood connectivity among soluble mediators. Connectivity power networks were assembled using a clustered layout to identify the connection between chemokines, pro-inflammatory/regulatory cytokines, and growth factors with significant correlation indices. The analyses of intrinsic connectivity scores revealed that subjects with seronegative status at baseline receiving a half dose displayed a more imbricate connectivity profile, with a higher number of connections amongst chemokines compared to the full dose at both timepoints (D28 and D28*) (**Figure 9**).

## 4 Discussion

This large trial evaluated a vaccine against SARS-CoV-2 and confirmed that a half dose of ChAdOx1 nCoV-19 is safe, effective, and immunogenic as the standard full dose. For the overall confirmed cases, the lower bound estimate under a 95% CrI for the effectiveness in the ChAd Half Dose group was within the 95% credibility interval estimated for the ChAd Full Dose group, indicating non-inferiority for this treatment.

A half dose regimen of the COVID-19 vaccine can double the availability of doses and cut costs in half. Considering the current scenario of having to maintain booster doses regularly to contain hospitalizations and deaths from COVID-19, it is relevant to know that a half dose regimen can confer same effectiveness as a full dose, to protect from infection, hospitalizations, and deaths.

The ChAdOx1n19 vaccine is of special interest to Brazil, as it is the vaccine with the lowest cost, about 2-4 times less than other immunizers. Furthermore, it is produced in Brazil, after the transfer of technology from AstraZeneca/Oxford to Fundação Oswaldo Cruz (Fiocruz), which allows for greater production for distribution in Brazil and other countries in Latin America and Africa, as has already occurred in other epidemics.

The study included 88.3% of all individuals of 18-49 years, from Viana City, in Brazil. A previous study showed that a low dose of ChAdOx 1 nCov-19 vaccine induced high titers of neutralizing antibodies, similar to the standard dose, at different ages. It was later shown that a low dose followed by a standard dose conferred greater efficacy than two standard doses (8, 9). Those preliminary studies highlighted the hypothesis that a half dose is immunogenic enough to induce cellular and humoral immune responses and to confer protection against SARS-CoV-2. This is the first study to provide evidence for using fractional doses as a strategy to face COVID-19. Khoury et al. found high correlation between neutralizing antibody response and efficacy against disease (15) to show that half or even quarter doses of some vaccines generate immune responses associated with high vaccine efficacy, as we could demonstrate in this trial.

The main limitations of the study were the lack of randomization and unavailability of a placebo group. The design of the study had to take into consideration the opportunity to perform an interventional trial to study the effectiveness of a half dose of ChAdOx1 when vaccines were available and being distributed in that population.

Despite this limitation, it was possible to confirm that the effectiveness of half dose is non-inferior to full dose for preventing new cases. Effectiveness of ChAdOx1 was estimated around 70% to prevent new cases (9). A retrospective longitudinal study of 75,919,840 Brazilian vaccinees from January to July 2021 evaluated the effectiveness of ChAdOx1 (Vaxzevria) or Coronavac (16). Individuals fully vaccinated (≥14 days after the second dose) with ChAdOx1 had a 78.1% (95% CI: 77.2–79.0) lower risk of developing symptomatic SARS-CoV-2 infection. In the elderly Brazilian population, the effectiveness of the two-dose schedule was 77.9% (95% CI: 69.2–84.2) against COVID-19, 87.6% (95% CI: 78.2–92.9) against hospitalization, and 93.6% (95% CI: 81.9–97.7) against death (17).

There were differences between the ChAd Full Dose and ChAd Half Dose groups according to age and sex distribution; however, the analysis considered random effects and specifically linked the same groups for either vaccinated with a half dose and full dose.

The fact that not all symptomatic cases took the PCR test is another limitation of the study, but we did analysis considering only those cases confirmed by RT-PCR showing non-inferiority between half dose and full dose.

Throughout the study period, the Delta variant was more frequent in most COVID-19 infections, according to the independent genomic surveillance reported in Brazil and Viana. The Delta variant was first identified in June, and by August corresponded with most of the sequences analyzed (data not shown).

We found higher levels of biomarkers after half dose after primary vaccination compared to full dose. We hypothesized that half dose induces later kinetics of biomarkers compared to full dose, and therefore after 28 days, levels were higher in the half dose group. Unfortunately, we did not evaluate the kinetics of biomarkers. Probably, the peak of biomarkers of the full dose was earlier.

This hypothesis can be supported by the observation that the kinetics of biomarkers is earlier in vaccines with a viral vector compared to natural infection with lower levels of viral particles (18).

Beyond advantages based on cost-reduction and production benefits, there is a lower potential of adverse events using fractional half dose. In fact, we reported lower rate and duration of symptoms after half dose. In our study, reported local and systemic AEs were mild, in line with previously reported studies (8, 9, 19). A prospective observational safety study on ChAdOx1 nCoV-19 vaccine in the real-life showed that younger, female, hypertension, history of allergy, and hypothyroidism individuals had two to three times higher odds for adverse events (19). For population at more risk for adverse events, low dose could be safer. Ramasany et al. (8) found that a lower dose of vaccine was less reactogenic than the standard dose of vaccine across all age groups. The mixed pattern of immune response, with pro-inflammatory and regulatory biomarkers observed in our study, may be associated with a lower frequency and severity of AEs after half dose. Although it was not possible to confirm this hypothesis with this sample size, this remains a subject of interest for future studies, considering that severe adverse event as thrombocytopenia was rarely reported previously.

Spike binding and neutralizing antibodies have been proposed as a correlate of protection for COVID-19 vaccines (20). It has been reported that mRNA-1273-vaccinees with PRNT_50_ titers of 10, 100, and 1000 have estimated vaccine efficacies of 78%, 91%, and 96%, respectively (20). Our findings are in agreement with this proposal as we found a global serum conversion of 100%, considering the PRNT_50_ titer of 10 as the cut-off and overall effectiveness of 67% and 69% for subjects receiving a full dose or half dose, respectively.

In pre-immune individuals, titers after the second dose were lower than those after the first dose, after a full dose or half dose, suggesting that there may be some mechanism of cellular exhaustion. Further studies are necessary to investigate this phenomenon.

Although there are no defined cellular immunity correlates of protection against COVID-19 infection and the immunological thresholds required for vaccine efficacy remain undefined, a favorable immune profile induced by ChAdOx1 vaccine has been proposed (21). Here, we found that a half dose of ChAdOx1 elicited a strong systemic, polyfunctional, and balanced soluble mediator response. To provide a more detailed overview of SARS-CoV-2-specific cellular response, phenotypic and functional analyses of memory T and B cells are currently under investigation in the Viana study.

A limitation of this study was the use of an external comparison group for immunogenicity assessment, especially due to the predominance of females in the ChAd Full Dose subsample. The significant contribution of sex to modulating vaccine-induced immunity has gained attention over the last several years. Specifically, females typically develop higher antibody responses and experience more AEs following vaccination than males, regardless of age (20, 22). We performed analyzes stratified by sex. The geometric mean titers found were similar in men and women. Comparisons between the ChAd Full Dose and ChAd Half Dose groups reproduced those results found in the general analyses. Despite the significant biological and behavioral differences between males and females, systematic review and meta-analysis concluded no significant sex differences in the efficacy of the COVID-19 vaccines, especially in younger populations (23).

We included the same 18-49 age range for both groups. This is a homogeneous age interval for vaccine reactivity. We conducted additional analysis, and the Spearman correlation of antibody titers (IgG-S) with age was very low (r = 0.1703, p = 0.0192). Our study did not include elderly people because, in that period, that population had already been vaccinated.

Further analyses are required to investigate the usefulness of a half dose for homologous or heterologous boosting as well as the effectiveness in both children and elderly populations. Although laboratory analyses reported in this study were done in blinded assays, ongoing studies of cellular immunity in larger groups will provide relevant additional information about the similarities between half and full doses.

In conclusion, a half dose of ChAdOx1 nCoV-19 is safe, effective, immunogenic, and non-inferior to the full dose. The immune response in pre-immune individuals indicates that the half dose may be a booster dose schedule.

## Half Dose Chadox Study Group

*Hospital Universitário Cassiano Antônio Moraes, Universidade Federal do Espírito Santo (HUCAM-UFES), Rede de Hospitais Federais (EBSERH)*: Thayná Martins Gouveia, Beatriz Paoli Thompson, Karen Evelin Monlevade Lança, Gabriela Curto Cristianes Lacerda, João Pedro Gonçalves Lenzi, Sabrina de Souza Ramos, Felipe de Castro Pimentel, Ludimila Forechi, Thaís Ruchdeschel, João Pedro Moraes Miossi, Matheus Leite Rassele, Gabriel Smith Sobral Vieira, Laís Pasti, Allan Gonçalves Henriques, Maria Eduarda Morais Hibner Amaral, Alessandro Demoner Ramos, Heitor Filipe Surlo, Laura Gonçalves Rodrigues Aguiar, Luiza Lorenzoni Grillo, Matheus Pereira, Ramon Borge Rizzi, Sara Monteiro Muniz, Hully Cantão dos Santos, Thais Luma de Oliveira Roza, Adriana Santos Silva, Lunara Baptista Ferreira, Karina Lallemand, Ketty Lysie Libardi Lira Machado, Tania Queiroz Reuter Motta. *Vigilância Municipal de Saúde de Viana*: Jaquelini Jubini, Carla Cristina Moraes de Mattos, Maria Angélica Calegário Vieira. *Vigilância Estadual de Saúde, Secretaria de Estado da Saúde do Espírito Santo*: Danielle Grillo Pacheco Lyra, Cristiano Soares da Silva, Rodrigo Ribeiro Rodrigues, Luís Carlos Reblin, Orlei Cardoso. *Organização Pan-Americana da Saúde (OPAS)*: Lely Stella Guzmán Barrera, Jhader Pércio. *Fundação Oswaldo Cruz (FIOCRUZ)*: *IRR-Fiocruz Minas* - Ismael Artur da Costa Rocha, Roberta Oliveira Prado, Agnes Antônia Sampaio Pereira, Vitor Hugo Simões Miranda, Gláucia Diniz Alessio, Fernanda Fortes de Araújo, Elaine Speziali, Christiane Costa Pereira, Clarice Carvalho Alves, Kétyllen Reis Andrade de Carvalho, Anna Carolina Cançado Figueiredo, Liliane Martins dos Santos, Cristiana Couto Garcia, Nani Oliveira Carvalho, Laise Rodrigues Reis, Tâmilla Mayane Alves Fidelis dos Santos, Joaquim Pedro Brito-de-Souza, Camila Medeiros Costa, Isabela Natália Pascoal Campos do Vale, Priscilla Miranda Henriques, Poliane Silva Maciel, Thais Abdala Torres, Nathália Werneck Cézar de Oliveira, Gabriela de Oliveira, Luana Oliveira Borges Fernandes, Andreza Parreiras Gonçalves, Jesuanne Carla Silva Andrade, Ladson Lúcio Viana da Silva, Armanda Moreira Mattoso Barbosa, Maria Beatriz Martins Araújo, Bruna Luiza Fonte Boa Rocha, Lis Ribeiro do Valle Antonelli, Ana Carolina Campi-Azevedo, Vanessa Peruhype-Magalhães; *Laboratório de Tecnologia Virológica (LATEV), Bio-Manguinhos, Fiocruz-RJ* - Waleska Dias Schwarcz, Nathalia dos Santos Alves, Ingrid Siciliano Horbach, Ariane Faria de Souza, Brenda de Moura Dias, Bruno Pimenta Setatino, Caio Bidueira Denani.

## Data availability statement

The original contributions presented in the study are included in the article/supplementary material. Further inquiries can be directed to the corresponding author.

## Ethics statement

The studies involving human participants were reviewed and approved by National Research Ethics Committee (CONEP, Protocol No. 4.752.775/2021); and the Ethics Review Committee of the Pan American Health Organization (PAHOERC, Protocol No. 0367.02/2021). The patients/participants provided their written informed consent to participate in this study.

## Author contributions

VV, OM-F, LC, DV, NM, AT-C, and JM conceived and designed the research study. LN and CD composed the advisory committee. VV, OM-F, NM, AT-C, and JM were responsible for acquiring the financing. VV, OM-F, MG, AA, AT-C, JM, IM, LD, SM, and CSG conducted the experiments. CSG acquired the data. VV, OM-F, LC, DV, JM, IM, LD, and SM analyzed and interpreted the data. VV, OM-F, LC, DV, SL, AA, LN, CD, AT-C, JM, and SM wrote and revised the manuscript. All authors reviewed, read, and approved the final version of the manuscript. All authors contributed to the article and approved the submitted version.

## Funding

The study was supported by Instituto de Ensino, Pesquisa e Inovação (ICEPI)/Secretaria de Estado da Saúde do Espírito Santo (SESA), Ministério da Saúde do Brasil, Fundação Oswaldo Cruz (FIOCRUZ), Hospital Universitário Cassiano Antônio Moraes da Universidade Federal do Espírito Santo (HUCAM-UFES), Empresa Brasileira de Serviços Hospitalares (EBSERH), Prefeitura Municipal de Viana, and Organização Pan-Americanca da Saúde (OPAS). The study was carried out by students and professors enrolled at the Post-graduation courses: Saúde Coletiva (PPGSC/UFES), Ciências da Saúde (IRR/FIOCRUZ), and Ciências da Saúde (PPGCIS/UFAM), all supported by the Coordenação de Aperfeiçoamento de Pessoal de Nível Superior (CAPES).

## Acknowledgments

The authors thank the Program for Technological Development in Tools for Health-RPT-FIOCRUZ for using the flow cytometry facilities and Laboratório de Inovação em Saúde (LAIS) from Universidade Federal do Rio Grande Norte (UFRN) for developing the digital platform. OM-F, LC, DV, CD, AT-C, and JM received PQ fellowships from CNPq, and OM-F and AT-C are research fellows from FAPEAM (PVN-II, PRÓ-ESTADO Program #005/2019. 

## Conflict of interest

Authors OM-F, LC, DV, SL, AA, and AT-C are staff members of FIOCRUZ, responsible for manufacturing the ChAdOx1 nCoV-19 COVID-19 vaccine.

The remaining authors declare that the research was conducted in the absence of any commercial or financial relationships that could be construed as a potential conflict of interest.

## Publisher’s note

All claims expressed in this article are solely those of the authors and do not necessarily represent those of their affiliated organizations, or those of the publisher, the editors and the reviewers. Any product that may be evaluated in this article, or claim that may be made by its manufacturer, is not guaranteed or endorsed by the publisher.
